# Cancer risk factors among people living with HIV/AIDS in China: a systematic review and meta-analysis

**DOI:** 10.1038/s41598-017-05138-x

**Published:** 2017-07-07

**Authors:** Zi-Yi Jin, Xing Liu, Ying-Ying Ding, Zuo-Feng Zhang, Na He

**Affiliations:** 1 Department of Epidemiology, School of Public Health, and the Key Laboratory of Public Health Safety of Ministry of Education, Fudan University, Shanghai, China; 2 Department of Epidemiology, Fielding School of Public Health, University of California, Los Angeles, Los Angeles, USA

## Abstract

Cancer is a leading cause of death among people living with HIV/AIDS (PLWHA). We conducted a systematic review and meta-analysis to evaluate prevalence of cancer risk factors among Chinese PLWHA based on 102 articles. Random effects meta-analysis was used to calculate the summary prevalence estimate (sPrev) and 95% confidence interval (CI) for each cancer risk factor by demographic group. Overall, the sPrev for each risk factor among Chinese PLWHA was: 41.1% (95% CI: 35.3–46.9%) for current smoking; 30.3% (95% CI: 23.3–37.4%) for current alcohol consumption; 24.4% (95% CI: 14.7–30.2%) for overweight and obesity; 12.5% (95% CI: 10.6–14.3%) for hepatitis B virus infection; 29.1% (95% CI: 23.6–34.5%) for hepatitis C virus infection; 33.9% (95% CI: 24.3–43.5%) for high-risk human papillomavirus infection from cervical samples and 78.6% (95% CI: 69.4–87.7%) from anal samples; 2.7% (95% CI: 0.7–4.7%) for Epstein-Barr virus (EBV) immunoglobulin M (IgM) positivity, 94.7% (95% CI: 90.7–98.8%) for EBV IgG positivity and 25.6% (95% CI: 12.4–38.8%) for EBV DNA positivity; 14.9% (95% CI: 12.4–17.4%) for human herpes virus 8 infection. The prevalence of major cancer risk factors was high among PLWHA in China, suggesting an urgent need for interventions to reduce cancer risk in this high-risk group.

## Introduction

Highly active antiretroviral therapy (HAART) or combination antiretroviral therapy (cART) has drastically reduced mortality in people living with HIV/AIDS (PLWHA)^1–3^. For PLWHA, cancer is now one of the leading causes of death with higher incidence of both AIDS-defining cancers (ADCs) and non-AIDS-defining cancers (NADCs) compared with the general population^4–8^. In particular, NADCs have shown increasing incidence and account for a rising proportion of all cancers in this population^4^. China has implemented HAART nationally since 2003. In a Chinese cohort, substantially elevated standardized incidence ratios of NADCS and ADCs were observed, and cancers occurred at a younger age for PLWHA compared to the general population^9^. Accumulated evidence suggests that HIV-induced immunodeficiency and prevalent risk factors other than HIV infection independently contribute to the increased cancer risk among PLWHA^10, 11^. The major risk factors associated with leading causes of cancer deaths include tobacco use (associated with lung, colorectal, stomach, and liver cancer), alcohol consumption (colorectal and liver cancer), overweight/obesity (breast and colorectal cancer), and viral infections (liver, stomach, and cervical cancer)^12, 13^. Numerous independent studies have reported prevalence of cancer risk factors among PLWHA in China. However, the researches vary in time and geographic areas, and do not provide a comprehensive overview of cancer risk factors epidemics for PLWHA in China. We conducted a meta-analysis among PLWHA in China to describe the prevalence of major cancer risk factors including tobacco smoking, alcohol consumption, overweight and obesity, and cancer-related viral infections such as hepatitis B virus (HBV), hepatitis C virus (HCV), human papillomavirus (HPV), Epstein-Barr virus (EBV) and human herpes virus 8 (HHV8). Prevalence estimates among Chinese PLWHA were compared with the general population or uninfected comparison groups from the same study when available.

## Results

### The process of study selection

The process of selection of publications and the numbers of articles for each cancer risk factor are presented in Table 1. A total of 3,955 peer-reviewed research articles were identified from four databases: PubMed (219), CNKI (2,381), Wanfang Data (586), and CQVIP (769). We excluded 3,407 articles after screening titles and abstracts. Among the remaining 548 studies (101 in English and 447 in Chinese), we further excluded 446 studies during full-text review for the following reasons: 171 were studies with an irrelevant population, 138 had no prevalence estimate, 33 did not present original data, 44 were reviews, 39 had a small sample size (less than 100), and 21 were redundant articles. Finally, 102 eligible publications (35 in English and 67 in Chinese) contributed to the meta-analyses^14–115^. One additional study with unique data on HIV uninfected population was included for comparison analysis^116^.

**Table 1:** The process for selection of studies, by cancer risk factor.

Flow	Category	Cancer risk factor							
		Smoking	Alcohol Consumption	Overweight/obesity	Hepatitis B or C virus infection	Human papilloma virus infection	Epstein-Barr virus	Human herpes virus 8	Total
Identified articles^a^	PubMed	29	73	12	65	20	6	14	219
	CNKI	387	407	149	849	170	291	128	2381
	Wanfang	40	44	15	396	46	20	25	586
	CQVIP	75	68	43	486	74	1	22	769
	Total	531	592	219	1796	310	318	189	3955
Excluded after screening of title and abstract	Total	383	435	189	1661	267	298	174	3407
Full-text review	English article	10	29	5	28	16	2	11	101
	Chinese article	138	128	25	107	27	18	4	447
	Total	148	157	30	135	43	20	15	548
Excluded after full-text review with reasons for exclusion	No relevant population	29	62	2	67	6	1	4	171
	No prevalence estimate	63	45	7	14	2	7	0	138
	No original data	14	4	15	0	0	0	0	33
	Review	14	20	2	2	4	1	1	44
	Sample size < 100	7	6	1	6	13	1	5	39
	Redundant article	2	4	0	2	9	3	1	21
	Total	129	141	27	91	34	13	11	446
Eligible articles	English article	1	6	1	17	6	1	2	34
	Chinese article	18	10	2	27	3	6	2	68
	Total	19	16	3	44	9	7	4	102
Contributed to the meta-analysis^b^		26	32	6	HBV:29; HCV:42	9	9	4	102

^a^Date searched from database inception to February 29, 2016 for all cancer risk factors, except for hepatitis B or C virus infection (January 1, 2013–February 29, 2016).

^b^Including eligible articles identified from both the specific risk factor and other risk factors.

### Characteristics of eligible publications

Table 2 shows the characteristics of the eligible articles. Ninety-two cross-sectional studies, 7 prospective cohort studies^38, 52, 60, 80, 94, 100, 101^, 2 retrospective cohort studies^68, 79^ and 1 intervention trial^36^ were included. Most studies (99) were based in clinics or hospitals for PLWHA recruitment, 2 were population-based^72, 73^ and 1 study sampled was recruited from jail^75^. The 102 eligible studies covered 20 provinces and 4 municipalities in China. The median sample size for PLWHA was 295 [interquartile range (IQR): 178–551]. Seventy-five articles reported the median or mean age, and the median of the median or mean age was 37.2(IQR: 34.0–41.0). Of the 95 articles reporting gender distribution, the median proportion of male was 72.6% (IQR: 61.3–86.9%). Of the 16 articles reporting the proportions of MSM, the median was 79.1% (IQR: 63.9–100.0%). Of the 23 articles reporting the proportions of IDU, the median was 74.8% (IQR: 40.4–100.0%). Of 26 articles reporting the proportions on ART, the median was 67.5% (IQR: 45.3–100.0%).

**Table 2:** Characteristics of eligible articles.

Author (Publication year)	Province	Calendar years	Age (Mean/Median)	N	Male (%)	MSM (%)	IDU (%)	ART (%)
Yao (2015)^14^	Shaanxi	…	35.5	535	93.5	71.8	…	100.0
Wei (2015)^15^	Jiangsu	2014–2014	39.2	276	91.7	…	2.5	100.0
Pu (2011)^16^	Guangxi	2008–2010	34.3	184	84.8	…	42.9	…
Xiao (2014)^17^	Anhui	2012–2012	44.5	801	59.9	…	…	100.0
Nurbiya (2014)^18^	Xinjiang	2013–2013	40.1	109	59.6	…	…	…
Zhang (2012)^19^	Shanxi	2004–2005	42.0	316	53.8	…	…	…
Luo (2014)^20^	Yunan	2012–2012	38.1	455	66.2	…	55.4	67.5
Bao (2015)^21^	Shanghai	2014–2014	38.3	284	94.0	…	…	…
Sun (2014)^22^	Liaoning	2010–2011	37.4	772	89.5	…	…	35.2
Cheng (2014)^23^	Beijing	2013–2013	34.0	504	91.7	…	…	…
Cheng (2012)^24^	Taiwan	2010–2010	31.0	305	100.0	100.0	…	47.5
Cheng (2014)^25^	Taiwan	2011–2011	32.9	230	100.0	78.7	…	…
Zhang (2014)^26^	Yunan	2009–…	34.0	301	0.0	…	…	64.1
Wu (2012)^27^	Taiwan	2008–2009	38.8	803	94.9	75.2	4.9	…
Luo (2014)^28^	Chongqing	2012–2013	38.3	124	75.0	…	8.1	54.0
Dong (2006)^29^	Guangxi	2005–2005	…	321	69.8	…	30.8	…
Li (2011)^30^	Shanghai	2005–2009	42.0	348	84.5	…	2.9	20.7
Zhou (2015)^31^	Yunan	…	59.8	201	…	…	…	…
Tsai (2014)^32^	Taiwan	2002–2006	37.3	320	90.6	62.8	…	94.4
Zhou (2015)^33^	Hunan	2010–2015	…	240	44.6	…	…	48.3
Jiang (2014)^34^	Anhui	2012–2012	49.0	261	55.6	…	…	100.0
Su (2010)^35^	Anhui	2006–…	40.4	153	43.8	…	…	…
Xie (2015)^36^	Heilongjiang	2010–2012	36.0	240	92.5	…	…	…
Zhao (2012)^37^	Guangxi	2010–2011	54.6	100	75.0	…	…	…
Lin (2015)^38^	Chongqing	2008–2011	48.6	188	66.5	36.2	61.7	62.8
Zhang (2008)^39^	Anhui	2005–…	41.9	219	48.4	…	…	…
Gu (2013)^40^	Sichuan	2005–2011	43.2	506	78.7	…	…	…
Wang (2011)^41^	Henan	2005–2009	45.2	162	50.0	…	…	…
Lin (2010)^42^	Shandong	2009–2009	37.9	324	61.4	…	…	100.0
Ge (2015)^43^	Zhejiang	…	42.4	123	83.7	…	…	…
Li (2014)^44^	Shandong	…–2013	…	184	92.9	…	…	…
Liu (2014)^45^	Henan	…	45.0	706	57.8	…	…	…
Li (2009)^46^	Beijing	2005–2006	…	179	60.9	…	…	…
Muessig (2014)^47^	Guangdong	2011–2011	31–40	721	61.3	…	…	100.0
Luo (2013)^48^	Yunan	2012–2012	38.1	455	66.2	…	55.4	67.5
Hu (2013)^49^	Beijing	2010–2011	30.4	212	100.0	100.0	…	…
Yang (2010)^50^	Sichuan	2007–2009	33.2	102	100.0	100.0	100.0	46.1
Jin (2014)^51^	Yunan	…	36.8	204	65.7	…	100.0	…
Yen (2012)^52^	Taiwan	2007–2010	40.0	194	83.0	…	100.0	…
Zhang (2013)^53^	Tianjin	2011–2012	42.1	100	89.0	…	…	…
Dong (2011)^54^	Guangxi	2010–2010	39.3	400	63.8	…	…	…
Zhou (2013)^55^	Guangxi	…	…	125	0.0	…	…	…
Pan (2015)^56^	Guangxi	2012–2014	…	158	72.2	…	100.0	…
Shen (2015)^57^	Yunan	…–2013	…	663	96.1	…	100.0	…
Dong (2015)^58^	Yunan	…	34.0	498	86.9	…	100.0	…
Zhou (2014)^59^	Zhejiang	2009–2012	38.5	572	72.6	…	…	…
Liu (2014)^60^	Sichuan	1995–2010	32.6	614	81.4	…	…	…
Zhang (2014)^61^	China	2010–2012	…	33861	66.5	…	…	…
Chen (2013)^62^	Yunan	2006–2008	39.0	978	78.0	…	…	…
Chen (2016)^63^	Sichuan	2011–2014	…	126	54.8	…	…	…
Wu (2015)^64^	Jiangsu	2010–2014	…	145	89.7	…	…	…
Pan (2015)^65^	Jiangxi	…	35.1	134	68.7	…	…	…
Ye (2013)^66^	Yunan	2005–2006	…	544	65.6	…	…	…
Li (2013)^67^	Chongqing	2009–2012	30–40	994	68.9	…	…	…
Pererdun (2015)^68^	Xinjiang	2005–2011	35.1	2357	54.5	…	…	…
Zhang (2013)^69^	Shandong	2000–2010	35.0	2021	62.7	…	…	…
Zhang (2014)^70^	Shaanxi	…	…	164	64.6	…	…	78.7
Zhao (2013)^71^	Hubei	2007–2010	38.6	356	73.6	…	…	29.2
Dong (2014)^72^	Sichuan	2010–2010	…	694	69.5	…	…	…
Liu (2014)^73^	Henan	…	29.1	164	55.5	…	…	…
Zhou (2014)^74^	Sichuan	2004–2012	…	1633	9.8	…	100.0	…
Hsieh (2014)^75^	Taiwan	2008–2010	36.4	297	85.5	…	100.0	…
Liu (2015)^76^	Xinjiang	…	…	129	…	…	100.0	…
Ng (2013)^77^	Taiwan	2006–2010	…	162	…	…	100.0	…
Zhang (2013)^78^	Guangdong	2006–2011	…	145	…	…	100.0	…
Li (2016)^79^	Multiple	2005–2014	34.0	1105	71.7	…	…	100.0
Peierdun (2015)^80^	Xinjiang	2006–2011	36.6	2252	54.2	…	…	…
Pan (2014)^81^	Zhejiang	2013–2013	31–40	321	84.7	…	…	…
Duan (2014)^82^	Jiangxi	2004–2012	42.7	2194	71.4	…	…	…
Tang (2013)^83^	Sichuan	2012–2013	…	378	…	…	…	…
Wang (2015)^84^	Anhui	2014–2014	37.0	122	50.0	…	…	100.0
He (2015)^85^	Sichuan	2012–2014	37.1	285	60.4	…	…	…
Liu (2013)^86^	Yunan	2008–2011	…	1025	…	…	…	…
Zheng (2013)^87^	Shanghai	2005–2010	39.7	1153	82.8	…	…	…
Zhang (2015)^88^	Shaanxi	2005–2013	36.0	1243	91.7	…	…	…
Shen (2013)^89^	Multiple	2009–2010	41.0	2040	75.7	…	…	…
Lee (2014)^90^	Taiwan	2009–2012	38.0	171	93.6	79.5	…	…
Cao (2013)^91^	Multiple	2009–2010	36.5	538	74.2	…	…	…
Feng (2013)^92^	Yunan	2009–2011	41.3	449	66.4	…	…	…
Li (2013)^93^	Sichuan	2006–2009	42.6	133	76.7	…	…	…
Kou (2013)^94^	Multiple	2009–2010	37.0	532	74.6	…	…	…
Wang (2014)^95^	Guizhou	2013–2013	…	204	74.0	…	…	…
Xiao (2014)^96^	Anhui	2011–2013	…	234	65.4	…	…	…
Liu (2014)^97^	Henan	2012–2013	…	1887	73.3	…	…	…
Zhao (2013)^98^	Hubei	2011–2012	…	332	100.0	100.0	…	…
An (2013)^99^	Multiple	2010–2012	31.5	513	100.0	100.0	…	…
Guo (2013)^100^	Guangdong	2009–2012	31.6	166	0.0	…	…	…
Zhang (2012)^101^	Hubei	2009–2011	41.0	293	0.0	…	…	…
Luo (2013)^102^	Multiple	2009–2010	…	182	0.0	…	…	…
Yu (2012)^103^	Taiwan	2010–2011	…	194	100.0	67.0	…	84.5
Li (2016)^104^	Multiple	…	32.2	104	100.0	100.0	…	…
Yang (2015)^105^	Hubei	2007–2014	37.6	157	72.6	…	…	…
Zhang (2014)^106^	Beijing	2005–2011	…	731	86.6	…	…	100.0
Hu (2014)^107^	Hubei	2010–2013	41.0	217	77.4	…	…	…
Wu (2012)^108^	Guangdong	2010–2011	36.5	257	72.8	…	…	…
Zhou (2011)^109^	Shanxi	2006–2006	…	175	64.6	…	…	…
Shi (2012)^110^	Yuan	2008–2010	35.0	245	66.5	1.6	43.7	40.8
He (2011)^111^	Multiple	2008–2009	30–39	1110	61.4	…	…	42.7
Yang (2009)^112^	Xinjiang	2006–2006	31.4	468	51.1	…	40.4	…
Li (2014)^113^	Yuan	2008–2010	…	245	…	…	…	…
Lee (2014)^114^	Shanxi	2004–2006	33.8	504	86.7	25.2	74.8	29.8
Yang (2013)^115^	Jiangsu	2012–2012	37.2	264	100.0	100.0	…	…

### Prevalence estimates for cancer risk factors

**Figure 1:**
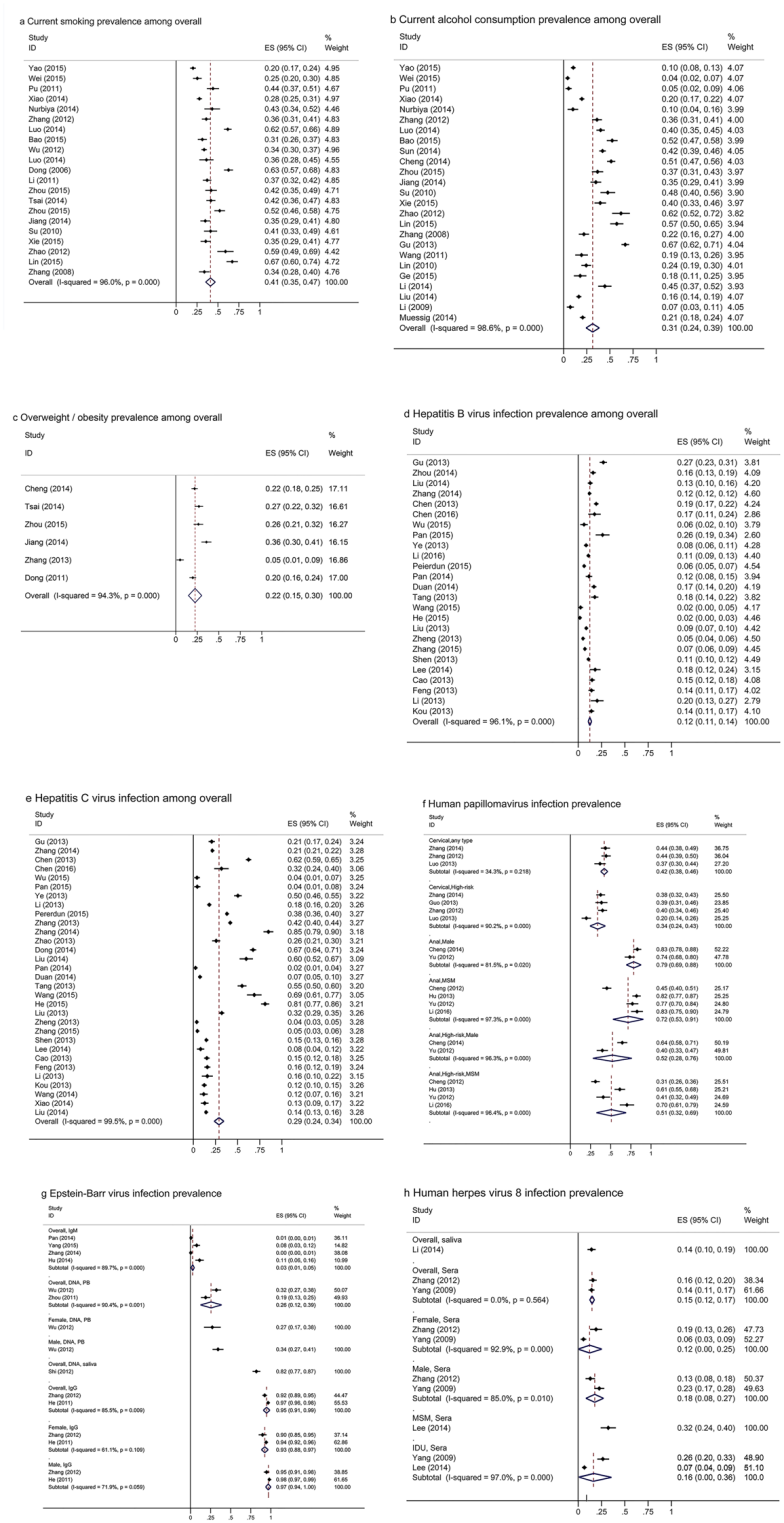
Selected forest plots showing the results of meta-analysis of cancer risk factor prevalence among persons living with HIV/AIDS. (**a**) Current smoking prevalence among overall; (**b**) Current alcohol consumption prevalence among overall; (**c**) Overweight/obesity prevalence among overall; (**d**) Hepatitis B virus infection prevalence among overall; (**e**) Hepatitis C virus infection prevalence among overall; (**f**) Human papillomavirus infection prevalence; (**g**) Epstein-Barr virus infection prevalence; (**h**) Human herpes virus 8 infection prevalence.

Table 3 presents the group-specific sPrev estimates for each cancer risk factor among PLWHA and the corresponding prevalence in the Chinese general population. Selected forest plots for important cancer risk factors are shown in Fig. 1. Moreover, Table 4 presents the results comparing the prevalence between PLWHA and HIV uninfected groups if data were available from the same studies.

**Table 3:** Pooled and single prevalence of cancer risk factors among persons living with HIV/AIDS by demographic group and prevalence in general population, in China.

Risk factor, demographic group	Prevalence (%) (95% CI)	I^2^ (%), *P*-value	N	References	Prevalence (%, 95% CI) in general population
**Smoking**					
Current					
Overall^a^	41.1 (35.3–46.9)	96.0, <0.001	21	14–21, 27–39	28.3 (27.2–29.4)^117, 119^
Female	3.4 (2.2–4.6)	0.0, 0.709	5	17–20, 26	2.5 (1.9–3.0)^117, 119^
Male^b^	63.2 (39.7–86.8)	98.9, <0.001	5	17–20, 25	53.3 (51.4–55.2)^117, 119^
MSM	47.0 (41.4–52.7)	—	1	24	
Ever					
Overall	51.9 (42.8–61.1)	95.9, <0.001	5	20–23, 27	
Female	8.0 (4.8–11.2)	—	1	26	
Former					
Overall	13.9 (0.3–27.6)	98.6, <0.001	3	20, 21, 27	15.3 (14.3–16.4)^117, 119^
Female	5.1 (2.5–7.7)	—	1	26	25.6 (21.0–30.3)^117, 119^
**Alcohol Consumption**					
Current					
Overall	30.3 (23.3–37.4)	98.6, <0.001	25	14–23, 33–47	28.8^119^
Female	3.3 (1.2–5.4)	67.8, 0.045	3	17, 19, 20	9.4^119^
Male	26.9 (18.5–35.4)	91.8, <0.001	5	17–20, 25	47.6^119^
MSM	35.7 (18.9–52.5)	95.1, <0.001	3	24, 49, 50	
IDU	13.1 (8.3–17.9)	51.5, 0.151	2	51, 52	
Ever					
Overall	64.3 (60.8–67.7)	0.0, 0.577	2	21, 48	36.4^119^
Female	16.9 (11.0–22.8)	—	1	48	14.5^119^
Male	89.7 (86.3–93.1)	—	1	48	57.7^119^
Former					
Overall	17.8 (3.6–32.0)	96.5, <0.001	2	20, 21	
Female	64.3 (56.7–71.9)	—	1	20	
Male	5.0 (2.5–7.4)	—	1	20	
**Overweight and obesity**					
Overall	22.4 (14.7–30.2)	94.3, <0.001	6	23, 32–34, 53, 54	42.6^119^
**Hepatitis B virus infection (HBsAg)**					
Overall	12.5 (10.6–14.3)	96.1, <0.001	25	40, 59–66, 79–94	7.2 (6.7–7.7)^120^
Female	10.4 (8.1–12.8)	66.2, 0.003	9	59–66, 79	5.7^120^
Male	13.4 (11.0–15.8)	89.0, <0.001	10	57, 59–66, 79	8.6^120^
MSM	12.7 (9.1–16.2)	—	1	98	
IDU	32.8 (0.0–70.7)	99.7, <0.001	3	57, 58, 75	
**Hepatitis C virus infection**					
Overall	29.1 (23.6–34.5)	99.5, <0.001	31	40, 61–73, 81–97	0.43 (0.33–0.53)^121^
Female	29.9 (23.2–36.6)	98.4, <0.001	16	55–57, 61–74	0.40 (0.27–0.54)^121^
Male	47.1 (32.2–62)	99.8, <0.001	15	56, 57, 61–74	0.46 (0.31–0.61)^121^
MSM	2.0 (1.1–3.0)	0.0, 0.873	2	98, 99	
IDU	83.4 (63.8–100.0)	99.8, <0.001	6	57, 58, 74–76, 78	
**Human papillomavirus infection**					
Cervical					
Any type	42.0 (37.7–46.3)	34.3, 0.218	3	26, 101, 102	9.9–27.5^123^
High-risk types	33.9 (24.3–43.5)	90.2, <0.001	4	26, 100–102	21.1 (20.8–21.3)^122^
Anal					
Any type, Male	78.6 (69.4–87.7)	81.5, 0.020	2	25, 103	17.8 (16.2–19.3)^124^
Any type, MSM	71.7 (52.8–90.6)	97.3, <0.001	4	24, 49, 103, 104	
High-risk, Male	52.1 (27.9–76.2)	96.3, <0.001	2	25, 103	6.4 (5.4–7.3)^124^
High-risk, MSM	50.7 (32.3–69.2)	96.4, <0.001	4	24, 49, 103, 104	
**Epstein-Barr virus**					
Overall, IgM	2.7 (0.7–4.7)	89.7, <0.001	4	81, 105–107	
Overall, DNA, PB	25.6 (12.4–38.8)	90.4, 0.001	2	108, 109	
Female, DNA, PB	27.1 (16.7–37.6)	—	1	108	
Male, DNA, PB	34.2 (27.4–41.0)	—	1	108	
Overall, DNA, saliva	82.0 (77.2–86.8)	—	1	110	
Overall, IgG	94.7 (90.7–98.8)	85.5, 0.009	2	19, 111	97.6^125^
Female, IgG	92.5 (88.3–96.7)	61.1, 0.109	2	19, 111	
Male, IgG	96.8 (93.6–100.0)	71.9, 0.059	2	19, 111	
**Human herpes virus 8**					
Overall, saliva	14.3 (9.9–18.7)	—	1	113	
Overall, Sera	14.9 (12.4–17.4)	0.0, 0.564	2	19, 112	11.3 (7.2–15.5)^126^
Female, Sera	12.1 (0.0–25.3)	92.9, <0.001	2	19, 112	
Male, Sera	17.7 (8.3–27.2)	85, 0.010	2	19, 112	
MSM, Sera	32.3 (24.2–40.4)	—	1	114	
IDU, Sera	16.3 (0.0–35.7)	97.0, <0.001	2	112, 114	

^a^‘Overall’ means PLWHA were not restricted by sex or HIV transmission category.

^b^‘Male’ means PLWHA were not restricted by HIV transmission category.

**Table 4:** Comparison of prevalence of cancer risk factors between HIV infected and uninfected groups from the same study.

Risk factor, study	Study design	Matching conditions	Category, Demographic group^a^	HIV infected		HIV uninfected		*P*-value
				N	Prevalence (%)	N	Prevalence (%)	
**Smoking**								
Su (2010)^35^	Cross-sectional	Age, gender	Current, Overall	153	41.2	153	25.5	0.004
Cheng (2012)^24^	Cross-sectional	None	Current, MSM	304	47.0	100	32.0	0.008
**Alcohol Consumption**								
Su (2010)^35^	Cross-sectional	Sex, age	Current, Overall	153	47.7	153	54.9	0.208
Jin (2014)^51^	Cross-sectional	None	Current, IDU	204	15.7	202	19.3	0.337
Jin (2014)^51^ ^b^	Cross-sectional	Age, gender, education, and ethnicity	Current, IDU	204	15.7	201	12.4	0.883
Yen (2012)^52^	Prospective cohort	None	Current, IDU	194	10.8	1250	10.6	0.938
Cheng (2012)^24^	Cross-sectional	None	Current, MSM	305	33.8	100	50.0	0.004
Hu (2013)^49^	Cross-sectional	None	Current, MSM	212	19.8	453	26.5	0.062
**Hepatitis B virus infection**								
Hsieh (2014)^75^	Cross-sectional	None	IDU	297	18.9	265	11.3	0.013
**Hepatitis C virus infection**								
Hsieh (2014)^75^	Cross-sectional	None	IDU	297	98.7	265	83.0	<0.001
**Human papillomavirus infection**								
Zhang (2012)^101^	Prospective cohort	None	Cervical-any type, Female	293	44.4	200	20.0	<0.001
Luo (2013)^102^	Cross-sectional	None	Cervical-any type, Female	182	36.8	300	19.3	<0.001
Guo (2013)^100^	Prospective cohort	None	Cervical-high-risk, Female	166	38.6	476	10.5	<0.001
Zhang (2012)^101^	Prospective cohort	None	Cervical-high-risk, Female	293	39.9	200	17.5	<0.001
Luo (2013)^102^	Cross-sectional	None	Cervical-high-risk, Female	182	19.8	300	1.7	<0.001
Cheng (2012)^24^	Cross-sectional	None	Anal-any type, MSM	305	45.2	100	18.0	<0.001
Hu (2013)^49^	Cross-sectional	None	Anal-any type, MSM	212	82.1	459	57.5	<0.001
Li (2016)^104^	Cross-sectional	None	Anal-any type, MSM	104	82.7	718	62.8	<0.001
Cheng (2012)^24^	Cross-sectional	None	Anal-high-risk, MSM	305	31.1	100	13.0	<0.001
Hu (2013)^49^	Cross-sectional	None	Anal-high-risk, MSM	212	61.3	459	39.7	<0.001
Li (2016)^104^	Cross-sectional	None	Anal-high-risk, MSM	104	70.2	718	48.2	<0.001
**Epstein-Barr virus**								
Shi (2012)^110^	Cross-sectional	Age, gender	Saliva (EB-DNA), Overall	245	82.0	30	30.0	<0.001
**Human herpes virus 8**								
Li (2014)^113^	Cross-sectional	None	Saliva, Overall	245	14.3	30	0.0	<0.001
Zhang (2011)^116^	Cross-sectional	None	Sera, Overall	305	16.4	315	4.8	<0.001
Lee (2014)^114^	Cross-sectional	None	Sera, MSM	127	32.3	104	15.4	<0.001
Lee (2014)^114^	Cross-sectional	None	Sera, IDU	377	6.6	176	1.1	<0.001

^a^Demographic versus between HIV infected and uninfected group: Overall VS General, Female VS General female, MSM VS MSM, and IDU VS IDU.

^b^HIV uninfected controls were enrolled from non-IDU group.

### Smoking

A total of twenty-six articles reported prevalence of smoking among PLWHA. Overall, the sPrev for current smoking was 41.1% (95% CI: 35.3–46.9%*, I* ^2^ = 96.0%) in PLWHA based on the meta-analysis of 21 studies. The sPrev of current smoking was 63.2% (95% CI: 39.7–86.8%) for male PLWHA and was 3.4% (95% CI: 2.2–4.6%) for females, based on the 5 studies with gender-specific prevalence. Overall, the sPrev of ever and former smoking was 51.9% (95% CI: 42.8–61.1%) based on 5 studies and 13.9% (95% CI: 0.3–27.6%) based on 3 studies, respectively (Table 3). Significantly higher smoking prevalence was observed in PLWHA than in HIV uninfected subjects when compared in the same studies (Table 4).

### Alcohol consumption

Thirty-two publications reported prevalence of alcohol consumption among PLWHA. Overall, the sPrev for current alcohol consumption was 30.3% (95% CI: 23.3–37.4%*, I* ^2^ = 98.6%) based on the meta-analysis of 25 studies. Gender-specific estimates showed that the sPrev of current alcohol consumption was 26.9% (95% CI: 18.5–35.4%, *I* ^2^ = 91.8%) in male PLWHA based on 5 studies and was 3.3% (95% CI: 1.2–5.4%, *I* ^2^ = 67.8%) in females based on 3 studies. MSM presented a current drinking sPrev of 35.7% (95% CI: 18.9–52.5%) based on 3 studies while IDUs presented a current drinking sPrev of 13.1% (95% CI: 8.3–17.9%) based on 2 studies. Overall, the sPrev of ever and former alcohol drinking was 64.3% (95% CI: 60.8–67.7%) and 17.8% (95% CI: 3.6–32.0%), respectively (Table 3). Six studies compared the prevalence of alcohol consumption between PLWHA and HIV negative controls, 5 of which reported no significant difference while 1 reported a lower prevalence in HIV positive MSM than in HIV negatives (33.8% vs. 50.0%, *P* = 0.004) (Table 4).

### Overweight and obesity

Six studies reported the prevalence of overweight and obesity (BMI ≧ 24 kg/m^2^) among PLWHA, with a sPrev of 24.4% (95% CI: 14.7–30.2%*, I* ^2^ = 94.3%) (Table 3).

### Hepatitis B virus infection

Twenty-nine articles reported the prevalence of HBV infection defined as HBsAg seropositivity in PLWHA. The sPrev of HBsAg was 12.5% (95% CI: 10.6–14.3%*, I* ^2^ = 96.1%), 10.4% (95% CI: 8.1–12.8%*, I* ^2^ = 66.2%) and 13.4% (95% CI: 11.0–15.8%, *I* ^2^ = 89.0%) in overall, female and male groups, respectively. The highest sPrev of HBsAg was among IDUs (32.8%, 95% CI: 0.0–70.7%*, I* ^2^ = 99.7%) (Table 3). In a study reporting comparisons with HIV uninfected group, HBsAg prevalence in PLWHA was also higher in IDU group (18.9% vs. 11.3%, *P* = 0.013) (Table 4).

### Hepatitis C virus infection

Forty-two publications reported the prevalence of HCV infection defined as HCV antibody seropositivity. Based on the meta-analysis of 31 studies, the overall HCV sPrev was 29.1% (95% CI: 23.6–34.5%*, I* ^2^ = 99.5%). Male PLWHA showed a sPrev of 47.1% (95% CI: 32.2–62.0%*, I* ^2^ = 99.8%) based on 15 studies, while female PLWHA had a sPrev of 29.9% (95% CI: 23.2–36.6%*, I* ^2^ = 98.4%) based on 16 studies. The highest HCV prevalence was found in IDU group with a sPrev of 83.4% (95% CI: 63.8–100.0%*, I* ^2^ = 99.8%) (Table 3). Higher prevalence was found in HIV infected IDUs comparing to HIV-negative participants in the same study (Table 4).

### Human papillomavirus infection

Nine studies reported prevalence of HPV infection in PLWHA. The number of studies for each group-specific meta-analysis ranged from 2 to 4. For cervical samples, the HPV sPrev of both any type and high-risk type was 42.0% (95% CI: 37.7–46.3%*, I* ^2^ = 34.3%) and 33.9% (95% CI: 24.3–43.5%*, I* ^2^ = 90.2%), respectively. For anal samples, the sPrev among male PLWHA of any type of HPV was 78.6% (95% CI: 69.4–87.7%*, I* ^2^ = 81.5%), and the sPrev of high-risk type was 52.1% (95% CI: 27.9–76.2%*, I* ^2^ = 96.3%). Similar sPrev was observed among HIV positive MSM (Table 3). All comparisons between PLWHA and HIV uninfected group reported significantly higher prevalence of HPV in PLWHA from the same studies (Table 4).

### Epstein-Barr virus infection

Nine publications presented the prevalence of Epstein-Barr virus infection. The number of studies for meta-analysis ranged from 1 to 4 when stratified by EBV testing method and demographic groups. Immunoglobulin M (IgM) was tested in 4 studies and the sPrev was 2.7% (95% CI: 0.7–4.7%*, I* ^2^ = 89.7%). IgG was tested in 2 studies, and the sPrev in overall, female and male groups was 94.7% (95% CI: 90.7–98.8%*, I* ^2^ = 85.5%), 92.5% (95% CI: 88.3–96.7%*, I* ^2^ = 61.1%) and 96.8% (95% CI: 93.6–100.0%*, I* ^2^ = 71.9%), respectively. DNA from peripheral blood was tested in 2 studies, and the overall sPrev was 25.6% (95% CI: 12.4–38.8%*, I* ^2^ = 90.4%). One study reported the prevalence of 27.1% (95% CI: 16.7–37.6%) in female and 34.2% (95% CI: 27.4–41.0%) in male PLWHA for EBV DNA positivity (Table 3). One study tested DNA in saliva from PLWHA and the prevalence was 82.0% (95% CI: 77.2–86.8%), which was higher than the prevalence in uninfected comparison group (30.0%) (Table 4).

### Human herpes virus 8 infection

Four articles reported the prevalence of HHV8 infection in PLWHA, of which 3 studies had serum antibody and one had saliva DNA tested. The prevalence of saliva DNA positivity was 14.3% (95% CI: 12.4–17.4%). For serum antibody, the sPrev in overall, female, male and IDU groups was 14.9% (95% CI: 12.4–17.4%*, I* ^2^ = 0.0%), 12.1% (95% CI: 0.0–25.3%*, I* ^2^ = 92.9%), 17.7% (95% CI: 8.3–27.2%*, I* ^2^ = 85.0%) and 16.3% (95% CI: 0.0–35.7%*, I* ^2^ = 97.0%), respectively. The highest serum HHV8 prevalence was among MSM (32.3%, 95% CI: 24.2–40.4%) (Table 3). Significantly higher prevalence was consistently found in PLWHA than in the HIV-negative comparison groups from the same studies (Table 4).

### Sensitivity analyses and publication bias

We recalculated each sPrev_omi_ for all studies except the “omitted” one in turn, with the results of full meta-analyses named as sPrev_all_. The influence was evaluated by |sPrev_omi_ - sPrev_all_|/sPrev_all_. All observed influences were less than 15% in our sensitivity analyses. Influences greater than 10% could be found in smoking among females (3.4% for all studies versus 3.1% for studies with higher potential for bias omitted) and males (63.2% vs. 55.4%), alcohol consumption among males (26.9% vs. 30.2%), overweight and obesity (22.4% vs. 25.8%), and HCV infection among IDUs (83.4% vs. 93.0%).

In our meta-analysis, substantial publication bias was significantly observed by the Egger test and/or the Begg test in estimating the overall sPrev of smoking (*P*-value for Egger test was 0.028, *P*-value for Begg test was 0.057), the overall sPrev of alcohol consumption (*P*_Egger_ = 0.002, *P*_Begg_ = 0.007), the overall sPrev of HBV infection (*P*_Egger_ = 0.791, *P*_Begg_ = 0.018), and the sPrev of HCV infection overall (*P*_Egger_ = 0.141, *P*_Begg_ = 0.014), in females (*P*_Egger_ = 0.015, *P*_Begg_ = 0.344), and in males (*P*_Egger_ = 0.095, *P*_Begg_ = 0.621).

## Discussion

To our knowledge, this is the first comprehensive meta-analysis to report the summary prevalence of cancer risk factors among PLWHA in China. We found that Chinese PLWHA had higher prevalence of smoking, alcohol consumption, and virus infections including HBV, HCV, HPV, EBV and HHV8, but had lower prevalence of overweight and obesity when compared with the HIV-negative participants from the same studies and the Chinese general population.

For such two modifiable behavioral risk factors as tobacco and alcohol use, Chinese PLWHA showed high prevalence of tobacco smoking and alcohol drinking overall and especially among males. For example, the smoking prevalence is 53.3% for general Chinese men^117^, while the sPrev is even higher, reaching 63.2% for male PLWHA. Such elevated smoking prevalence in HIV infected people was also found in independent Chinese studies comparing with HIV-negative participants^24, 35^, and in a meta-analysis in western countries with lower smoking prevalence in general population^118^. The sPrevs also varied by group-specific analyses, with certain subgroups at higher risk in tobacco and alcohol use. The overall sPrev of alcohol drinking is close to the prevalence from Chinese general population (30.3% vs. 28.8%)^119^ while MSM showed slightly higher sPrev (35.7%) and IDUs showed significantly lower estimate (13.1%). As group I carcinogens defined by IARC, tobacco smoking and alcohol consumption are not only associated with a series of human cancers, but also with other important chronic diseases. Based on our summarized estimates, the high prevalence of tobacco and alcohol use especially among male PLWHA suggested the need for an effective and more targeted intervention in smoking and drinking behaviors as well as a close observation of their roles in HIV and cancer disease progression to reduce morbidity and to improve quality of life in this aging population with prolonged life expectancy.

The prevalence of cancer-related viral infections, including HBV, HCV, HPV, EBV and HHV8 infection, are significantly higher in PLWHA than in general Chinese population^120–126^, shown by the results from both meta-analyses and independent studies. By demographic group, 10.4–13.4% of PLWHA were HBsAg positive, and 29.1–47.1% were HCV antibody positive. Moreover, the highest prevalence of HBV (32.8%) and HCV (83.4%) infection were both found in IDUs, probably due to the shared transmission route with HIV. For female PLWHA, the sPrev of both any type of HPV (42.0%) and high-risk type of HPV (33.9%) from cervical samples were higher than the prevalence in Chinese general population aged 15–60 years^122, 123^. Moreover, we found that in anal samples collected from male PLWHA, the HPV prevalence was very high no matter whether they were MSM or not. These observations call attention since HPV infection is also a risk factor for anal cancer. The high seropositivity of EBV-IgG was close to the prevalence reported earlier in the Chinese general population (97.6%)^125^. Although the latter statistic might be limited to experimental conditions and in need of update, it is believed that EBV infection is very common among adults^127^. Moreover, several studies suggested higher detection rate of EBV DNA among HIV-infected subjects compared to HIV-negatives, suggesting potentially more active viral application in immune-suppressed population^110, 128, 129^. Last but not least, the overall seroprevalence of HHV8 infection was 14.9%, higher than the prevalence in the Chinese general population^126^, with the highest prevalence found in MSM (32.3%, 95% CI: 24.2–40.4%).

The overall prevalence of overweight and obesity among PLWHA was 22.4% (95% CI: 14.7–30.2%), lower than the prevalence of 42.6% in Chinese general population^119^. This is similar with the observations in western countries that the obesity prevalence was lower in HIV population^118^. We need to further observe the change in weights in PLWHA to have a better understanding and balance in control of both the HIV wasting syndrome and risk for obesity-related cancers and chronic diseases.

A meta-analysis was recently published on prevalence of risk factors for NADCs among PLWHA in western high-income countries^118^, which, consistent with our analysis, also observed higher prevalence of cancer risk factors among PLWHA than the general population. However, the prevalence level seems to be different between PLWHA in western countries and in China. Compared with PLWHA in western countries, PLWHA in China seemed to have higher prevalence in alcohol consumption (30.3% versus 24.0%), HCV (29.1–47.1% versus 23.0–28.0%) and HBV (10.4–13.4% versus 4.0–5.0%) infection, but lower prevalence in smoking (41.1% versus 54.0%), overweight and obesity (22.4% versus 53.0%) and HPV infection (cervical high-risk types: 33.9% versus 46.0%, anal high-risk types: 52.1% versus 66.0%). Thus, the spectrum of cancers among PLWHA in China might be different from that in western countries and further investigations and interventions might be targeted to different high-risk groups.

Substantial heterogeneity across subgroups was significantly observed with *I* ^*2*^ values larger than 90%. This was expected since heterogeneity could be found in almost all ‘overall’ groups, because we could not know the proportion of genders or behavioral characteristics in these study populations across publications. So was the case in ‘male’ groups, which could have different proportions of heterosexual male, MSM and IDU. Meanwhile, extensive heterogeneity still existed in specific demographic groups such as female, MSMs and IDUs. This could be attributable to many potential factors including study design, sampling method, geographical regions, study period, age, ethnicity, the definition or method of measurement for risk factors, differences in CD4^+^ and the length of time in ART, etc^118, 130^. Insufficient number of studies has limited our further stratification analysis. However, random effects models were used to minimize the impact of heterogeneity on precision and provided rational approximations of pooled prevalence estimates in our meta-analyses. Sources of heterogeneity should be further explored in the future with more research for specific subgroups.

Several limitations in our study should be noted. First, there might be some information bias since the methods for measuring these risk factors might vary across studies. Most (69.2%) studies did not give a clear definition of smoking habits (e.g., ever smoking: have smoked at least 100 cigarettes in life; Current smoking: smoked within the past 30 days) or did not have a precise measurement for smoking dose or frequency, while only 50% of studies presented the range of time (past 30 days or a day) for current alcohol drinking. Although we tried to extract information from reference studies using standardized definitions, misclassification might still exist since not every study gave a clear definition for each risk factor. Also, the testable HPV subtypes varied across studies, and therefore, underestimation of HPV prevalence might have occurred in studies with fewer testable HPV subtypes. Second, the representativeness of our meta-analyses is compromised since it covered 24 but not all 31 provinces or municipalities in China, underscoring the need for expanded research and surveillance efforts on cancer risk factors among PLWHA all over the country. Third, comparability was not considered across different sources of data due to time lags among studies. Some risk factors may present a temporal trend, some may not. For those with an increasing or decreasing trend, it may have compromised the comparison. Fourth, as prevalence estimates of cancer risk factors can vary with characteristics including age, lack of matching for age and other factors also limited the validity of the comparisons between PLWHA and the general population. However, consistent results were still observed when stratified roughly by age where possible (Supplemental Table S1). Fifth, substantial publication bias was observed according to rigorous publication bias tests, and might be attributable to several factors, including the presence of extensive heterogeneity, which we have discussed above^131, 132^. The association between behavioral changes such as smoking and alcohol consumption and severity of HIV patients might lead to underestimation of prevalence of cancer risk factors among PLWHA.

Despite these limitations, the present study provides a broad overview of prevalence of cancer risk factors among PLWHA in China. Both in comparison with the Chinese general population and with the uninfected comparison groups, the studies reach a wide consensus on higher prevalence of cancer risk factors among PLWHA. Along with the management of HIV individuals and ART program, interventions to reduce cancer risk factors should also be implemented in this population. Cancer prevention measures in PLWHA should include smoking cessation, HBV and HCV treatment, vaccination against HBV and HPV, annual cervical and anal Pap tests and cancer screening according to relevant guidelines^4, 133–138^. Furthermore, a better understanding of cancer risk attributable to specific factors in different HIV infected groups is needed to develop strategies in preventing cancer.

## Methods

### Search strategy

We searched for peer-reviewed research articles published in English and Chinese from the following databases: PubMed, China National Knowledge Infrastructure (CNKI), Wanfang Data and Chinese Scientific Journals Fulltext Database (CQVIP). Using the Medical Subject Headings (MeSH) terms ‘China’ or ‘Taiwan’ with ‘HIV,’ ‘HIV Infections’ or ‘Acquired Immunodeficiency Syndrome’, the search also included MeSH terms for the specific cancer risk factors as follows: smoking (‘Smoking’ or ‘Tobacco Products’ or ‘Tobacco’ or ‘Tobacco Use’), alcohol consumption (‘Alcohol Drinking’ or ‘Alcoholism’ or ‘Alcoholic Intoxication’), overweight or obesity (‘Overweight’ or ‘Obesity’ or ‘Body Mass Index’ or ‘Body Weight’), HBV (‘Hepatitis B’), HCV (‘Hepatitis C’), HPV (‘Papillomavirus Infections’ or ‘Papillomaviridae’), EBV (‘Epstein-Barr Virus Infections’), and HHV8 (‘Herpesvirus 8, Human’). We also searched those MeSH terms and their abbreviations of each risk factor under the condition of ‘all fields’ in each database. Due to the large number of studies published on HBV and HCV infections among PLWHA in China, we restricted published data from January 1, 2013 to February 29, 2016 in order to use the latest published prevalence of HBV and HCV infections. For other cancer risk factors, data published from database inception to February 29, 2016 were included in the analyses.

### Study selection

One author (Jin Z-Y) identified relevant articles, excluding those with unrelated titles and abstracts before obtaining full-text references. Two independent authors (Jin Z-Y and Liu X) conducted full-text review. Cross-sectional, case-control, prospective or retrospective cohort and intervention trial studies were included. Studies with sample size greater than 100 were considered to have a sufficiently stable prevalence estimate to be qualified for inclusion in the study. Studies lacking original data for the numerator or denominator of the prevalence estimate were excluded. If information from one study had been published for more than once, we chose the publication with more detailed information such as prevalence estimates stratified by specific demographic groups, studies with a larger sample size, or those most recently updated.

### Data extraction

Data were extracted and organized using Microsoft Excel (Version 2010, Microsoft Corp., Redmond, WA, USA). For each risk factor, the number of PLWHA with known status was recorded as the denominator and the number of positive subjects was recorded as the numerator. The participants with unknown status were excluded from the calculation. If an article contained more than one of the mentioned risk factors, we kept all of them. If the PLWHA were stratified into different groups such as females, males, men who have sex with men (MSM) or injection drug users (IDU), we included data from each subgroup for prevalence estimate. The definitions of smoking and drinking status including “ever/never”, “former/current” were based on the descriptions in the original studies when a clear definition is not available. We also extracted prevalence estimates for HIV uninfected comparison groups if data were available from the same study. Other information including the first author’s name, year of publication, study site, original study design, sampling frame, calendar years of research, major information for participants including the mean or median age, percentage of males, MSM, IDU, treatment with antiretroviral therapy (ART), and the measurement of risk factors were also extracted.

We also extracted prevalence estimates for cancer risk factors among the general population from the most representative surveys. For tobacco smoking, alcohol consumption, overweight and obesity, prevalence estimates were extracted from a national survey, which was carried out in Chinese adults (aged 18 years and over with the median age in the range from 45 to 49 years) at 162 disease surveillance points (DSPs) from 31 provinces in 2010^117, 119^. For HBV and HCV infections, prevalence estimates were obtained from a national serosurvey in 2006, of which participants were local residents aged 1–59 years (the median age was in the range from 15 to 59 years for HBV and in the range from 15 to 20 years for HCV) living in 31 provinces with 160 DSPs^120, 121^. For HPV infection, prevalence estimates were acquired from a nationwide investigation of female subjects aged 15–60 years in 37 Chinese cities in 2012, a review (age ranging from 15 to 59 years) and a population-based study among men aged 25–65 years (the median age was 43 years)^122–124^. For EBV infection, the prevalence estimate was extracted from a 1989 study by Liu^125^. For HHV8 infection, the prevalence estimate was from a systematic review and meta-analysis published in 2012^126^.

### Statistical analysis

PLWHA were stratified into overall, female, male, MSM, and IDU demographic groups when such subgroups were available. The ‘Overall’ group represents PLWHA not restricted by gender or HIV transmission category (i.e., did not include a research on specific group by gender or HIV transmission category). Similarly, the ‘male’ group implies that PLWHA were not restricted by HIV transmission category. A meta-analysis of prevalence was performed for each risk factor by demographic group if there was more than one study included. Heterogeneity tests across subgroups were examined by Q-test (*P* < 0.1 refers to statistically significant heterogeneity) and *I* ^2^ values^139, 140^. Random effects models were used because of significant heterogeneity across subgroups^141, 142^. DerSimonian and Laird method (D-L) was applied to estimate the summary prevalence estimate (sPrev) and its 95% confidence interval (CI) for each risk factor^143^. If only one study was presented for the specific subgroup, we showed the single prevalence and its 95% CI. In sensitivity analyses, each sPrev was recalculated excluding studies evaluated as having higher potential for bias. Lastly, we performed a publication bias test when the meta-analysis included more than 10 studies^144, 145^. Publication bias was assessed through the Egger weighted regression and Begg rank correlation method (*P* < 0.1 represents statistically significant publication bias)^146, 147^. All the analyses were conducted by Stata 12.0 (Stata Corp LP).

## Electronic supplementary material

Supplementary Information
