# Supplementary material for: Cancer risk factors among people living with HIV/AIDS in China: a systematic review and meta-analysis
Source: Sci Rep. 2017 Jul 7;7:4890. doi: 10.1038/s41598-017-05138-x (PMC5501798; doi:10.1038/s41598-017-05138-x)
Supplement: Supplementary file 1 — Supplementary Information [file 41598_2017_5138_MOESM1_ESM.pdf]

# Cancer risk factors among people living with HIV/AIDS in China: a systematic review and meta-analysis


**Authors and Affiliations:**

Zi-Yi Jin[1 §], Xing Liu[1 §], Ying-Ying Ding[1], Zuo-Feng Zhang[2], Na He[1*]

1. Department of Epidemiology, School of Public Health, and the Key Laboratory of Public Health Safety of Ministry of Education, Fudan University, Shanghai, China ;
2. Department of Epidemiology, Fielding School of Public Health, University of California, Los Angeles, Los Angeles, U.S.A.

[§] Equally contributed first authors.

[*]**Corresponding author:** Na He, MD., PhD., Professor of Epidemiology, School of Public Health of Fudan University and The Key Laboratory of Public Health Safety of Ministry of Education (Fudan University) , Shanghai 200032, China. Tel/Fax: (86)-21-54237519; Email: nhe@fudan.edu.cn.

Supplemental Table S1. Pooled and single prevalence of cancer risk factors among persons living with HIV/AIDS and prevalence in general population by age, in China

| Risk factor, demographic group | Median/Mean age range (year) | Prevalence (%) (95%CI) | $I^2$ (%), *P*-value | N | References | General population [c] | |
|---|---|---|---|---|---|---|---|
| | | | | | | Age group (year) | Prevalence (%) [d] |
| **Current smoking** | | | | | | | |
| Overall [a] | 35.5-44.5 | 36.4 (30.8-42.1) | 94.7, <0.001 | 15 | [14-21,27,28,30,32,35,36,39] | 18-44 | 27.7 |
| | 48.6-59.8 | 50.5 (35.0-66.1) | 94.9, <0.001 | 4 | [31,34,37,38] | 45-59 | 31.4 |
| | | | | | | 60- | 25.2 |
| Female | 34.0-44.5 | 3.4 (2.2-4.6) | 0.0, 0.709 | 5 | [17-20, 26] | 18-44 | 1.6 |
| | | | | | | 45-59 | 2.9 |
| | | | | | | 60- | 5.0 |
| Male [b] | 32.9-44.5 | 63.2 (39.7-86.8) | 98.9, <0.001 | 5 | [17-20, 25] | 18-44 | 52.5 |
| | | | | | | 45-59 | 58.9 |
| | | | | | | 60- | 46.5 |
| **Current Drinking** | | | | | | | |
| Overall | 35.5-40.2 | 28.4 (19.5-37.3) | 98.9, <0.001 | 17 | [14-23,35,36,39,40,42,43,47] | 18-44 | 29.5 |
| | 48.6-54.6 | 37.8 (20.2-55.3) | 97.9, 0.000 | 5 | [34,37,38,41,45] | 45-59 | 31.1 |
| | | | | | | 60- | 22.4 |
| Female | 38.1-44.5 | 3.3 (1.2-5.4) | 67.8, 0.045 | 3 | [17,19,20] | 18-44 | 9.5 |
| | | | | | | 45-59 | 10.2 |
| | | | | | | 60- | 7.6 |
| Male | 32.9-44.5 | 26.9 (18.5-35.4) | 91.8, 0.000 | 5 | [17-20,25] | 18-44 | 48.5 |
| | | | | | | 45-59 | 51.3 |
| | | | | | | 60- | 37.9 |
| **Overweight and obesity** | | | | | | | |
| Overall | 34.0-42.1 | 18.3 (9.7-27.0) | 94.4, <0.001 | 5 | [23,32,53,54] | 18-44 | 37.6 |
| | 49.0 | 35.6 (29.8-41.4) | | 1 | [34] | 45-59 | 51.9 |
| | | | | | | 60- | 44.8 |
| **Hepatitis B virus infection (HBsAg)** | | | | | | | |
| Overall | 32.6-39.7 | 11.4 (8.6-14.1) | 95.9, <0.001 | 14 | [59,60,62,65,79,80,81,84,85,87,88,90,91,94] | 1-19 | 1.0-5.4 |
| | 41.0-43.2 | 17.6 (12.1-23.1) | 94.3, <0.001 | 5 | [40,82,89,92,93] | 20-29 | 10.5 |
| | | | | | | 30-39 | 8.6 |
| | | | | | | 40-49 | 8.5 |
| | | | | | | 50-59 | 8.9 |
| **Hepatitis C virus infection** | | | | | | | |
| Overall | 29.1-43.2 | 29.1 (23.6-34.5) | 99.5, <0.001 | 31 | [40,61-73,81-97] | 1-24 | 0.09-0.41 |
| | | | | | | 25-44 | 0.53-0.65 |
| | | | | | | 45-59 | 0.70-0.83 |
| Female | 29.1-40.0 | 29.9 (23.2-36.6) | 98.4, <0.001 | 16 | [55-57,61-74] | 1-24 | 0.07-0.29 |
| | | | | | | 25-44 | 0.48-0.74 |
| | | | | | | 45-59 | 0.67-0.75 |

| | | | | | | |
|---|---|---|---|---|---|---|
| Male | 29.1-40.0 | 47.1 (32.2-62.0) | 99.8, <0.001 | 15 [56,57,61-74] | 1-24 | 0.08-0.58 |
| | | | | | 25-44 | 0.42-0.76 |
| | | | | | 45-59 | 0.73-0.99 |
| **Human papillomavirus infection** | | | | | | |
| Cervical | | | | | | |
| Any type | 34.0-41.0 | 43.9 (39.9-47.9) | 0.0, 0.835 | 2 [26,101] | 15-59 | 9.9-27.5 |
| High-risk types | 34.0-41.0 | 38.7 (35.2-42.1) | 0.4, 0.836 | 3 [26,100,101] | 15-19 | 30.55 |
| | | | | | 20-29 | 22.17 |
| | | | | | 30-39 | 19.71 |
| | | | | | 40-49 | 20.55 |
| | | | | | 50-60 | 23.3 |
| Anal | | | | | | |
| Any type, Male | 32.9 | 83.0 (78.2-87.9) | - | 1 [25] | 25-65 | 17.8 |
| High-risk, Male | 32.9 | 64.3 (58.2-70.5) | - | 1 [25] | 25-65 | 6.4 |
| **Human herpes virus 8** | | | | | | |
| Overall, Sera | 31.4-42.0 | 14.9 (12.4-17.4) | 0.0, 0.564 | 2 [19,112] | - | 11.3 |

[a] 'Overall' means PLWHA were not restricted by sex or HIV transmission category.

[b] 'Male' means PLWHA were not restricted by HIV transmission category.

[c] The reference for each cancer risk factor is the same with that indicated in table 3, respectively.

[d] Some of the prevalence are presented as a range for combined age groups.
